# Editorial: State-Of-The-Art Fluorescence Image-Guided Surgery: Current and Future Developments

**DOI:** 10.3389/fonc.2021.776832

**Published:** 2021-10-22

**Authors:** John R. Benson, Fijs W. B. van Leeuwen, Tomoharu Sugie

**Affiliations:** ^1^ Cambridge Breast Unit, Addenbrooke’s Hospital, Cambridge and School of Medicine, Anglia Ruskin University, Cambridge, United Kingdom; ^2^ Interventional Molecular Imaging Laboratory, Department of Radiology, Leiden University Medical Center, Leiden, Netherlands; ^3^ Breast Surgery, Kansai Medical University Hospital, Hirakata, Japan

**Keywords:** fluorescence, indocyanine green, 5-aminolevulinic acid (5-ALA), fluorescent-guided surgery, sentinel node mapping, angiography, image-guided surgery

Fluorescent-guided surgery (FGS) is an intra-operative imaging modality with a potentially broad application and range of clinical utility. It provides an additional visual dimension to white light and represents an extension of pre-operative radiological investigations with real-time imaging during an operation (open or minimally invasive procedure). The main clinical usage relates to lymphatic mapping or evaluation of blood flow in the context of tissue perfusion (1). In addition, FGS has shown promise as a method for more accurately localizing and delineating solid tumors and detecting smaller impalpable tumor foci not evident pre-operatively or visualized with conventional white light-based technologies at the time of surgery.

Indocyanine green (ICG) is the most commonly used fluorophore that is considered clinically safe and has exclusive FDA approval (1). These near-infrared fluorescence properties initially led to development of dedicated intra-operative imaging systems that allow blood flow measurements during cardiac and transplantation surgery. These perfusion applications have been successfully extended to parathyroid surgery, partial nephrectomies, and anastomosis. Clinical usage has subsequently expanded with notable applications that rely on local dye accumulation. Examples are identification of hepatic lesions and sentinel lymph node (SLN) biopsy where ICG appears safe and of low toxicity (2, 3). With for example SLN biopsy, fluorescence guidance permits visualization of lymphatic channels and nodes, yielding high levels of concordance (>90%) for SLN detection by ICG in relation to blue dye or radioisotope (RI) (4–8). It remains unclear if ICG as a sole tracer can replace standard tracers (RI and blue dye) and whether a combination of ICG with either RI or blue dye optimizes performance parameters (6, 7). Hybrid approaches using shared tracer platforms may offer a convenient alternative to combinations of individual techniques (**Figure 1A**) (9). Notwithstanding potential advantages, there are drawbacks of fluorescent imaging that include significant signal attenuation by overlaying tissue and interference from ambient white light (surgical lights) during open surgery. Fluorescent imaging of tumors can be challenging where distinction between malignant and benign tissues relies on differential sensitivity. Development of tumor-targeting strategies for FGS can improve the chance of achieving radical resection of tumors with clear surgical margins and inclusion of occult foci of disease. Such approaches have been described using antibodies, peptides, and small molecules (10–12) which all offer exciting opportunities for refining the field of FGS.

**Figure 1:**
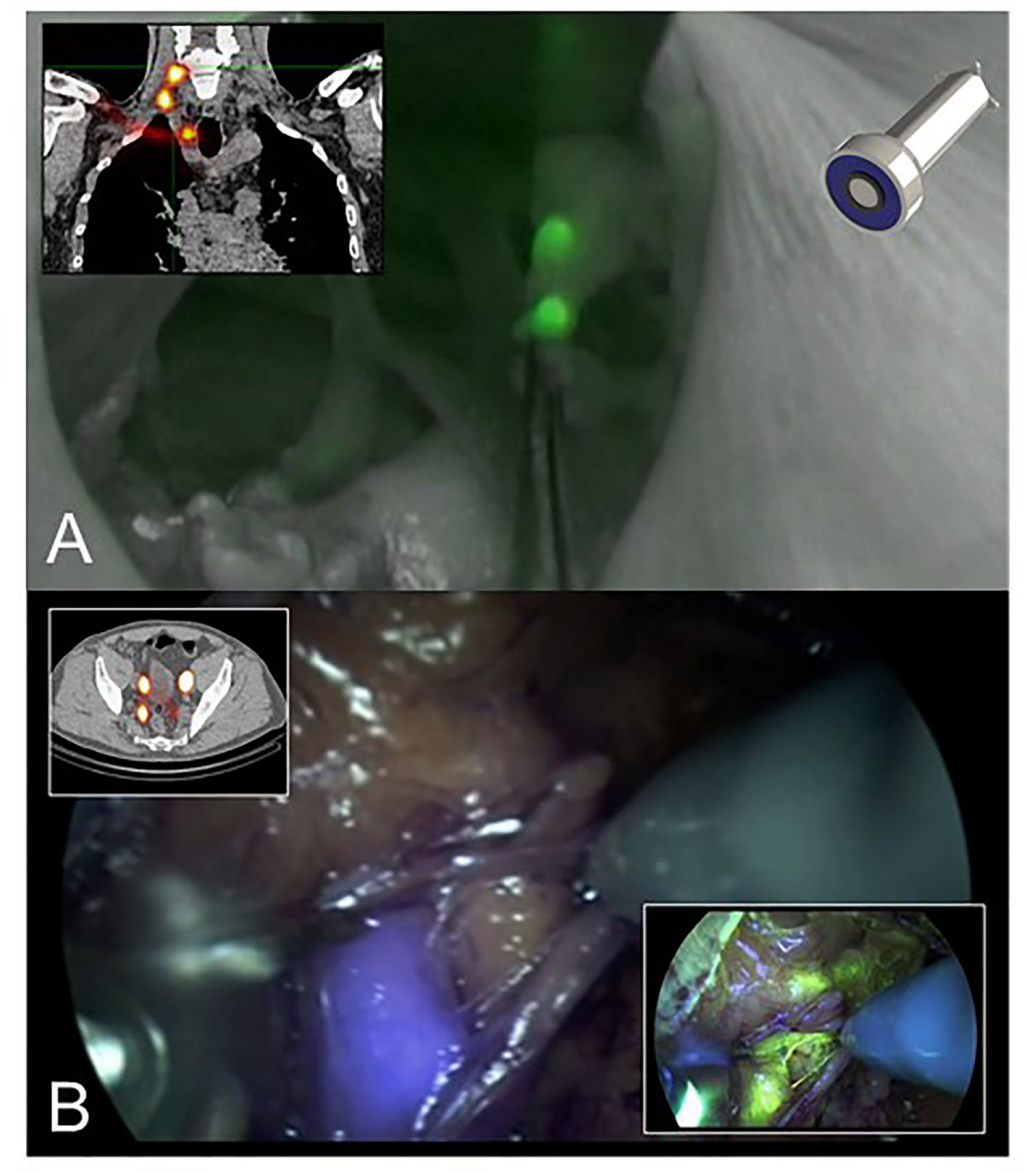
**(A)** The hybrid tracer ICG-99mTc-nanocolloid helps allying intraoperative fluorescence imaging with preoperative SPECT/CT data. This example shows SLN-biopsy in the head-and-neck region that was performed with the ambient lights on in the operation room (9). **(B)** Multi-color fluorescence imaging using the hybrid tracer ICG-99mTc-nanocolloid (preoperative SPECT/CT and blue) and fluorescein (yellow). This example shows robot-assisted SLN-biopsy in prostate cancer.

This series of research papers discusses current applications of FGS surgery and strategies for widening clinical usage of this technology during both conventional and minimally invasive surgery. A shared principle is the visual augmentation of molecular feedback that provides an adjunct to tactile feedback in open surgery and compensates for lack of this with laparoscopic or robotic procedures.

Nakamura and colleagues report on a medical imaging projection system (MIPS) that provides a continuous real-time projection of fluorescence mapping onto the surgical field and overcomes the problem of periodic dimming of theatre lights. This novel system avoids the need to shift visual focus from direct viewing of the surgical field under white light to the TV monitor. Initial studies revealed comparable nodal identification rates to standard NIR methodology but relatively low node positivity rate (13) that have also been observed in this follow-on study (7%). Further work with larger numbers of patients is required to clarify the accuracy of MIPS in terms of false negative rates and for SLN biopsy following neoadjuvant chemotherapy for clinically node negative patients.

Utility for lymphatic mapping and detection of nodal metastases in breast and other cancers has dominated FGS literature in the field of surgical oncology. However, an important translational challenge is use of this technology to enhance the visualization of solid tumors in real-time during surgery; surgical extirpation remains the primary management option for most solid tumors and is potentially curative when complete removal of the index lesion and any satellite foci of tumor is achieved with clear surgical margins. In addition to their therapeutic role, monoclonal antibodies can be re-purposed and used to image tumors after being linked to a fluorophore in a bioconjugate structure. Vargas and colleagues describe how clinical studies with immunoconjugates provide ‘proof of principle’ for the concept of selective receptor binding and clinical feasibility of tumor-specific FGS for cancer treatment. However, these agents have limited specificity and it may be necessary to wait up to 7 days from injection before there is sufficient tumor contrast to proceed with FGS. This is attributable to slow clearance of these large molecules from non-target tissues and distinction between tumor and normal tissues may be further hindered by interactions between these monoclonal antibodies and immune-effector cells that produce confounding fluorescent signals. A possible solution to this problem is use of low molecular weight (LMW) agents, such as peptides or small molecules that retain a high receptor affinity but possess an improved pharmacokinetic profile. Six LMW agents are discussed that can potentially improve intra-operative visualization and identify foci of tumor that would otherwise have been undetected (PPV 90 – 95%) (14). Apart from promoting accurate tumor staging and margin negative resections, this FGS study suggests there is also a potential to detect widespread malignancy prior to cytoreductive surgery with adoption of a palliative approach rather than any radical and potentially morbid surgical procedure.

There are some important challenges for receptor-targeted FGS before adoption into routine clinical practice; these include uncertainties in optimum quantitative evaluation of drug performance parameters along with confounding imaging factors. Furthermore, most commercial FGS devices have been optimized for ICG imaging in terms of spectral and sensitivity requirements and these may differ for tumor-specific probes and other fluorescent bio-conjugates. Development of newer cancer markers must ensure the presence of high affinity targeting motifs to guarantee high tumor selectivity and minimize non-specific interactions within normal tissues. This may be facilitated by a net-neutral charge that will promote elimination from non-malignant tissue (15).

Ultimately FGS techniques should improve clinical outcomes by reducing local recurrence through more precise surgery with negative resection margins. Kaibori and colleagues review the clinical application of NIR fluorescence imaging for hepatic resection in liver surgery. These authors have developed a novel nanoparticle incorporating ICG as a signaling agent that is biocompatible and can potentially assess hepatic anatomy and function as well as acting as a therapeutic agent for drug delivery (so-called ‘theranostics’). The ICG lactosome relies on increased vascular permeability of cancerous tissues that retain these particles to permit FGS with tumor demarcation or anti-tumor effect of a therapeutic molecule that uses the lactosome as a carrier. Fluorescence imaging with either ICG or the porphyrin precursor (5-aminolevulinic acid (5-ALA) can improve accuracy through real-time visualization of hepatic tissue during surgery. Furthermore, ICG fluorescence can detect fibrosis within non-cancerous liver parenchyma and indicate severity. ICG has a higher sensitivity for detection of smaller tumors near the surface of the liver, although is associated with lower levels of specificity; by contrast, 5-ALA imaging showed a lower sensitivity but a higher specificity. Optimum imaging of superficial liver tumors might eventually be achieved through use of a combination of ICG and 5-ALA that employs the concept of multi-wavelength fluorescence imaging; the latter can be applied to other scenarios including imaging for SLN biopsy (**Figure 1B**) (16).

Formal anatomical liver resection is greatly assisted with techniques of FGS that can accurately (>90%) delineate segments and sub-segments during laparoscopic surgery in both cirrhotic and non-fibrotic livers, but careful planning is essential with appropriate simulation and selection of fluorescent imaging method (17).

There are several important applications of fluorescence imaging in surgical oncology not directly related to tumor location and adequacy of resection, but nonetheless can improve clinical outcomes for patients. Luo and colleagues have examined the role of ICG fluorescence angiography (ICG-FA) in prevention of major anastomotic leakage after minimally invasive surgery (MIS) for a range of esophageal malignancies. This relies on use of intra-operative fluorescent imaging to assess perfusion of the gastric conduit prior to definitive anastomosis. ICG-FA might allow more accurate and objective evaluation of blood flow in the anastomotic zone without reliance on visual inspection and detection of vascular pulsation or peristalsis. Larger studies with standardized surgical techniques are required to confirm these benefits of ICG-FA in terms of reducing anastomotic leakage (18, 19).

A number of vendors now supply laparoscopic systems that incorporate an NIR imaging options an example being integration of the firefly camera in the da Vinci robotic platform. Moreover, technologies for dynamic visualization of the gastric conduit are now available and provide more reliable information on adequacy of perfusion. Hence employment of real time intra-operative evaluation may reduce the frequency and severity of surgical complications, although the majority of anastomotic leaks do not lead to secondary surgery.

## Author Contributions

Original draft preparation: JB. Review and editing: FL and TS. All authors contributed to the article and approved the submitted version.

## Conflict of Interest

The authors declare that the research was conducted in the absence of any commercial or financial relationships that could be construed as a potential conflict of interest.

## Publisher’s Note

All claims expressed in this article are solely those of the authors and do not necessarily represent those of their affiliated organizations, or those of the publisher, the editors and the reviewers. Any product that may be evaluated in this article, or claim that may be made by its manufacturer, is not guaranteed or endorsed by the publisher.
